# Gender differences in nutritional status and determinants among infants (6–11 m): a cross-sectional study in two regions in Ethiopia

**DOI:** 10.1186/s12889-022-12772-2

**Published:** 2022-02-26

**Authors:** Aregash Samuel, Saskia J. M. Osendarp, Edith J. M. Feskens, Azeb Lelisa, Abdulaziz Adish, Amha Kebede, Inge D. Brouwer

**Affiliations:** 1 Ethiopian Public Health Institute (EPHI), Patriots Street, P.O. Box 1242 or 5645, Addis Ababa, Ethiopia; 2 Division of Human Nutrition and Health, Wageningen University, P. O. Box 17, 6700AA Wageningen, The Netherlands; 3 The Micronutrient Forum, 1201 Eye St NW. 10th Floor, Washington, District of Columbia, 20005-3915 USA; 4 Nutrition International Ethiopia, Nutrition International C/O Ethiopia-Canada Cooperation Office (CIDA-ECCO), Nifas Silk - Lafto Sub City, Kebele 04, H. No. 161/01, Addis Ababa, Ethiopia

**Keywords:** Gender, Determinants, Nutritional status, Stunting, Infants, Young children, Growth, Ethiopia

## Abstract

**Background:**

A limited number of studies suggest that boys may have a higher risk of stunting than girls in low-income countries. Little is known about the causes of these gender differences. The objective of the study was to assess gender differences in nutritional status and its determinants among infants in Ethiopia.

**Methods:**

We analyzed data for 2036 children (6–11 months old) collected as the baseline for a multiple micronutrient powders effectiveness study in two regions of Ethiopia in March–April 2015. Child, mother, and household characteristics were investigated as determinants of stunting and wasting. Multiple logistic regression models were used separately for boys and girls to check for gender differences while adjusting for confounders. The study is registered at http://www.clinicaltrials.gov/ with the clinical trials identifier of NCT02479815.

**Results:**

Stunting and wasting prevalence is significantly higher among boys compared to girls, 18.7 vs 10.7% and 7.9 vs 5.4%, respectively. Untimely initiation of breastfeeding, not-exclusive breastfeeding at the age of 6 months, region of residence, and low maternal education are significant predictors of stunting in boys. Untimely introduction to complementary food and low consumption of legumes/nuts are significant predictors of stunting in both boys and girls, and low egg consumption only in girls. Region of residence and age of the mother are significant determinants of wasting in both sexes. Analysis of interaction terms for stunting, however, shows no differences in predictors between boys and girls; only for untimely initiation of breastfeeding do the results for boys (OR 1.46; 95%CI 1.02,2.08) and girls (OR 0.88; 95%CI 0.55,1.41) tend to be different (*p* = 0.12).

**Conclusion:**

In Ethiopia, boys are more malnourished than girls. Exclusive breastfeeding and adequate dietary diversity of complementary feeding are important determinants of stunting in boys and girls. There are no clear gender interactions for the main determinants of stunting and wasting. These findings suggest that appropriate gender-sensitive guidance on optimum infant and young child feeding practices is needed.

**Supplementary Information:**

The online version contains supplementary material available at 10.1186/s12889-022-12772-2.

## Background

Globally, stunting – an indicator of chronic undernutrition – affects at least 149.2 million children under the age of 5 years [1]. The Ethiopia Demographic and Health Survey (DHS) 2016 reports that, despite some improvements in the last 16 years, Ethiopia still displays high rates of childhood malnutrition, with 38% of Ethiopian children under 5 years of age being stunted and 10% being wasted[2]. Child linear growth is the measure of chronic malnutrition.

Epidemiological studies demonstrate that stunting is frequently associated with repeated exposure to poor sanitation and hygiene; and individual factors such as a child’s gender, poor economic conditions [3, 4], child morbidity [4, 5], and inadequate infant and young child feeding (IYCF) practices [6] have been identified as immediate causes of child stunting. The studies also show that wasting or thinness is generally associated with recent illness and/or food deprivation [3], presence of infectious disease [4], and inappropriate complementary feeding [7]. For example, a too-early introduction of solid foods before 6 months of age has a significant association with long-term deterioration of physical growth [7, 8].

Recent studies from several countries worldwide show a higher prevalence of stunting in boys compared to girls [9–11]. In Senegal and Guatemala, the observed gender difference in stunting prevalence is attributed to differential feeding practices, with boys starting complementary feeding at an earlier age, i.e. 2–3 months of age, thereby reducing the period of exclusive breastfeeding [9, 11]. A study from South West Uganda reports that stunted children are significantly less likely to be introduced to complementary food (CF) at an appropriate age [10].

Little is known about the existence of gender differences in nutritional status and underlying IYCF practices in Ethiopian infants. The current health promotion activities do not take gender issues into account. Thus, our study aims to compare the prevalence and potential determinants of stunting and wasting between boys and girls. IYCF practices, dietary intake, and maternal and household characteristics are the main determinants investigated. The determinants of wasting as an important indicator of acute undernutrition in young children are also compared between boys and girls.

## Methods

This cross-sectional study was performed using the baseline data for a large effectiveness study on the use of multiple micronutrient powders (MNPs) within a local CF program on iron status, morbidity, and children’s growth. Methods and findings of the MNP effectiveness study are described in detail elsewhere [12].

### Study area and population

The baseline data collection took place in the predetermined nine districts from Oromiya and Southern Nations, Nationalities, and Peoples (SNNP) regions of Ethiopia between March and April 2015. Both regions were selected because of their similar characteristics in food security, child health, and nutritional status; explicitly stunting (37% and 39%) and wasting (10.6% and 6.0%) in Oromiya and SNNP regions respectively [2], and infant feeding practices [13].

The study population consisted of young children 6–11 months of age [12]. The study intended to assess whether there will be a difference in IYCF practices between boys and girls, particularly in the introduction of solid, semi-solid, or soft foods which usually need to be initiated at 6–8 months [12]. This difference will lead to a difference in nutritional status. In addition, the objective of the intervention study was to assess the effectiveness as well as risks and benefits of a low-dose (6 mg iron per serving every other day) iron Micronutrient Powders (MNP) on iron status, morbidity, and growth of Ethiopian infants and young children [12]. We chose this age group to ensure that all children continued to be eligible to receive the program intervention for the entire duration of the follow-up. Details on the study site and sample selection are described elsewhere [12].

#### Operational definition

**Stunting** is defined as low height-for-age and it results from chronic undernutrition.

**Wasting** is defined as low weight-for-height. It often indicates recent and severe weight loss, although it can also persist for a long time.

#### Sample selection

We used a three-stage sampling for sample selection. At the first stage, intervention and non-intervention districts were purposively selected which were 3 intervention districts and 3 non-intervention districts which make 6 districts per region in total 12. Out of these 12 districts, three districts were excluded based on the status of the grain-bank program leaving nine districts for the study. Once the districts were chosen, the sub-district (second stage) within the district were selected using inclusion criteria [12]. A total of 35 sub-districts were identified. At the third stage, the selection of households within the sampled sub-districts was performed using criteria including a child 6–11 months, a child not having any serious illness that changes his/her food intake, and a mother willing to stay in the sub-district during the study period. Before screening for admission, all children below 1-year age in the selected sub-districts were listed by Health Extension Workers. Using the criteria, 65 eligible children (6–11 months) per sub-district; a total of 2356 children were included.

A total of 2356 children from 35 sub-districts/clusters (the smallest administrative unit in Ethiopia) of 9 districts were screened and admitted to the study [12]. A child could participate in the study if he/she was ≥ 6 and < 12 months old on the recruitment day and living in one of the selected sub-districts. Participating children also had to be free from chronic conditions such as metabolic or neurological disorders that might impact their health (e.g. mental retardation). Exclusion criteria included the presence of serious disabilities that would affect normal growth and development. In addition, children with a severe or protracted illness for which continuous medication is required and children with severe undernutrition (weight-length-z score (WLZ) < -3 SD) were excluded from the study and referred to the nearest health facility. In total, 320 children were excluded, and the analyses were performed on data for 2036 children.

### Data collection and measurements

#### Procedures

Three data collectors were assigned to work on a study area of three sub-districts as a field team. Thirty-six data collectors and six field supervisors were trained in the administration of questionnaires (IYCF, 24 h. recall, and morbidity) and anthropometric measurements, including standardization of data collectors (see supplemental file). After nine days of training on methodological procedures and quality assurance, the questionnaires were tested in a pilot group and adapted based on the received feedback from the survey team. The questionnaires were translated into local languages (Oromifa and Amharic) and back-translated to English to ensure the quality of the translation. The data collectors’ measurements were standardized to ensure that the inter-observer variability was within tolerable limits. Supervisors received additional training on teamwork and monitoring and supervising the data collection process. The field teams were provided with training and data collection manuals, a chart for calculating age in months, an event calendar, and a World Health Organization (WHO) classification table for Weight for Height (WHZ) to identify severely malnourished children. Data were collected at the sub-districts’ health posts by interviewing the child’s mother/primary caregiver and taking anthropometric measurements. The supervisors monitored the measurements to ensure the quality of the data.

Anthropometric measurements (length and weight) were taken following standard procedure [14]. Weight was measured using the UNICEF Seca 874 U electronic scales (UNICEF Supply Division, Copenhagen, Denmark) with 100 g precision calibrated daily with a known weight, and height was measured on UNICEF’s standard measuring board (precision of 0.1 cm). All children were measured lying down. Measurements were taken in duplicate and repeated a third time if the difference between the first two was more than 0.5 cm or 0.5 kg. IYCF practices and morbidity status were assessed using a questionnaire based on WHO recommendations to collect data for the IYCF indicators [15]. A 1-day non-quantified 24 h dietary recall was collected, including information on the source of food, method of preparation, and meal description to assign all ingredients to the respective food groups consumed by the child in the previous 24 h period.

### Data processing and analysis

#### Data processing

All the questionnaires were manually checked for completeness before data entry. Data were coded in duplicate and analyzed using SPSS (Version 22.0 for Windows, IBM, New York, USA). The data were cleaned for inconsistencies and missing values. If inconsistencies and missing values could not be resolved by checking the original questionnaires, those data were excluded from further analysis. Children’s age was entered as the date of birth provided by the caregivers. Length-for-age z scores (LAZ) and WLZ z scores were determined using the WHO Anthro software version 3.2.2 [16] based on the WHO reference population (2006). Stunting was defined as LAZ < -2 of the standard deviation (SD) and wasting was WLZ z scores < -2 SD. IYCF indicators were calculated following the UNICEF guidelines [17]. For this study, we defined as ‘timely introduced to CF’ a) children aged 6–8 months who were fed breast milk and had had at least one solid or semi-solid food the previous day or b) children aged 9–11 months who had a recall age of the first introduction of CF between 6 and 8 months of age. The seven food groups described by the WHO [17] were used to classify foods consumed, namely: 1) grains, roots, and tubers; 2) legumes and nuts; 3) dairy products; 4) flesh foods; 5) eggs; 6) vitamin A-rich fruits and vegetables; 7) other fruits and vegetables. Minimum dietary diversity was defined as the consumption of four or more food groups from the seven food groups [17]. Minimum meal frequency was defined as the consumption of 2 or more (at age 6–8 months), 3 or more (at age 9–23 months) solid or semi-solid feeds for breastfeeding children, or 4 or more solid or semi-solid or milk feeds for non-breastfeeding children at age 6–23 months [17]. A minimum acceptable diet was defined as a combination of minimum dietary diversity and meal frequency [17]. Basic drinking water and adequate sanitation facilities were defined according to UNICEF and WHO’s joint monitoring programme water, sanitation, and hygiene (WASH) targets and indicators post-2015 [18]. Full anthropometric data (age or length or weight) were missing for about 8% of children and the WLZ z score of some of the children was below entry point (< = -3.0). Thus, children with incomplete data on nutritional status and the flagged cases were therefore excluded from our analyses.

#### Statistical analysis

All continuous data were checked for normality by investigating the skewness and kurtosis of the distribution. For the linear regression, we used a graph of standardized residuals against each of the predictor variables in the regression models. We also used graphs to test the homogeneity of variance of the residuals by plotting the residuals versus predicted values.

Child characteristics, i.e. age, height, weight, LAZ z score, WLZ z score, stunting, wasting, and IYCF indicators, mother/caregiver characteristics including mother’s age, marital status, maternal education and occupation, and household characteristics, i.e. WASH indicators and region of residence, were compared between boys and girls using Chi-square tests for categorical variables and Student t-tests for continuous variables.

Binary logistic regression was used to investigate the association between potential determinants vs stunting and wasting. Variables associated with the outcome at *p* < 0.2 were selected for multiple logistic regression, stratified by gender, and adjusted for age, sub-district*/* cluster, and mother’s characteristics. The interactions between each variable and gender were tested with cross-product terms. For continuous variables (LAZ z score and WLZ z score), multiple linear regression was performed including independent variables associated with the outcome in unadjusted analyses at *p* < 0.2. A *p*-value of < 0.05 was considered significant. For the tests of interaction terms, a *p*-value of < 0.2 was considered relevant [19].

## Results

The study participants’ (*n* = 2036) characteristics are summarized in Table 1. The average (± SD) age of children was 8.2 ± 1.7 months. An almost equal proportion of male (51.5%) and female (48.5%) children was included. Socio-demographic characteristics were not different between boys and girls. The average age of mothers was 25.4 ± 5.7 years. Almost half of the mothers were illiterate; among literate mothers, a majority (43.8%) had attended primary school (grades 1–8) only. Most households had adequate sanitation facilities and basic drinking water sources.

**Table 1:** Selected characteristics of young children in Oromiya and SNNP^a^ regions of Ethiopia, 2015

Characteristics	Total population	Boys		Girls	*p*-value^††^
	*n* = 2036	*n* = 1049		*n* = 987	
Region, Oromiya %	47.6	53.7		46.3	0.069
**Child characteristics**					
Child’s age in months, mean ± SD	8.2 ± 1.7	8.2 ± 1.7	8.2 ± 1.7		0.459
6–8 n (%)	113 (54.7)	562 (53.6)	551 (55.8)		0.327
9–1.9 n (%)	923 (45.3)	487 (46.4)	436 (44.2)		
Male child, n (%)	1049 (51.5)				
Height, mean ± SD, cm	68.4 ± 3.8	68.9 ± 3.8	67.9 ± 3.8		< 0.001
Weight, mean ± SD, kg	7.72 ± 1.1	7.9 ± 1.1	7.5 ± 1.0		< 0.001
Length-for-age z score (LAZ), mean ± SD	-0.65 ± 1.4	-0.86 ± 1.4	-0.43 ± 1.3		< 0.001
Stunted (< -2SD) %	14.8	18.7	10.7		< 0.001
Severely stunted (< -3SD) %	3.9	6.0	1.7		< 0.001
Weight-for-length z score (WLZ), mean ± SD	-0.37 ± 1.1	-0.43 ± 1.1	-0.30 ± 1.1		0.011
Wasted (< -2SD) %	6.7	7.9	5.4		0.026
Diarrhea (prior 1 wk) %	28.3	28.2	28.4		0.961
Common cold or flu (prior 1 wk) %	25.5	26.0	25.0		0.612
**Mother’s characteristics**					
Mother’s age in years, mean (SD)	25.4 ± 5.7	25.5 ± 5.9		25.4 ± 5.4	0.903
Mother’s educational status %					
Illiterate/none–formal education	48.4	48.8		48.0	0.374
Grades 1–8	43.8	42.8		44.8	
Grade 9 and above	7.8	8.4		7.2	
Marital status %					
Married	96.4	96.3		96.6	0.812
Single/separated/divorced/widowed	3.6	3.7		3.4	
Mother’s occupation %					
Housewife/live with family	80.9	80.4		81.6	0.499
Working mother^b^	19.1	19.6		18.4	
**Household characteristics**^**c**^					
WASH indicators					
Adequate sanitation %					
Adequate ^d^	93.9	94.3		93.5	0.517
Drinking water ^f^					
Basic drinking achieved	92.7	93.2		92.1	0.349
Farmland ownership %	91.2	90.1		92.3	0.149

^††^ Chi-square tests were used for categorical variables and Student t-tests (sig 2-tailed) were used for continuous variables to compare the characteristics between boys and girls

^a^ Southern Nations, Nationalities, and Peoples

^b^ Working mother includes farmer, trader, merchant, civil servant, and teacher

^c^ *n* = 2023 (undefined *n* = 13)

^d^ Adequate sanitation includes a pit latrine with a superstructure, and a platform or squatting slab constructed of durable material; inadequate sanitation includes open pit, shared facilities of any type, no facilities, bush, or field [18]

^f^ Basic drinking water includes piped water with the subcategories public tap and private tap, protected spring, protected well, water from a borehole, water from a tanker truck, and rainwater; inadequate basic drinking water: surface water, river, unprotected spring [18]

**Fig. 1:**
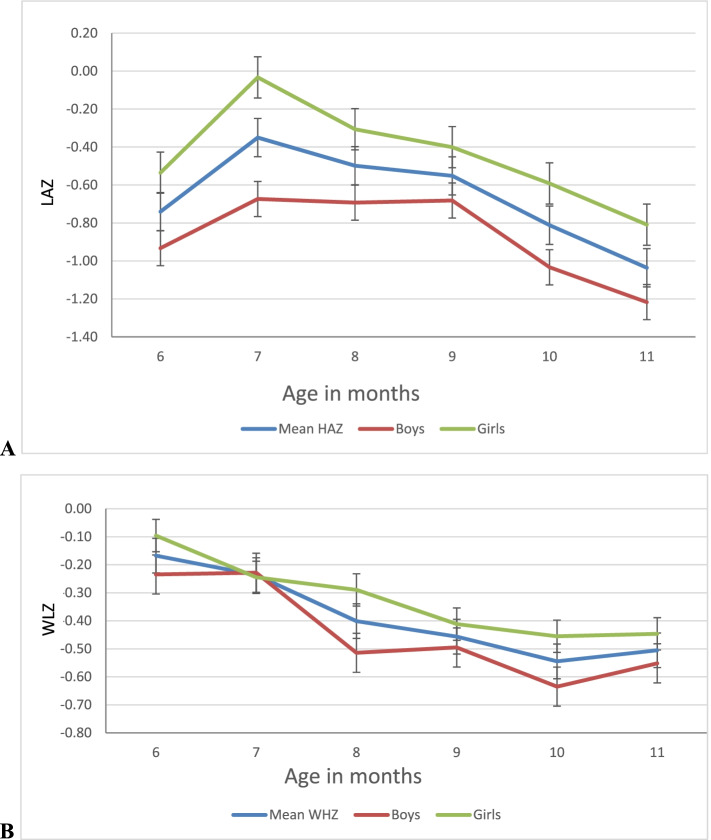
Stunting (**A**) and wasting (**B**) in Oromiya and SNNP†† regions of Ethiopia, 2015(*n* = 2036). †Error bars are defined using standard errors. †† Southern Nations, Nationalities, and Peoples

Stunting and wasting prevalence was significantly higher among boys than girls (18.7% vs 10.7%, *p* < 0.001, and 7.9% vs 5.4%, *p* < 0.026, respectively, Table 1), and girls had a significantly higher means of LAZ and WLZ z scores than boys. Stunting and wasting increased as the children’s age increased (Fig. 1, A and B).

In total, 28.3% of young children were reported as having diarrhea the week before the survey, and 25.5% were reported as having had a common cold or flu, with similar results for boys and girls (Table 1).

More than 75% of mothers started breastfeeding their new-born within one hour of birth (Table 2). Most children, 77% of boys and 78.2% of girls were exclusively breastfed during the first 6 months of life. Only 5.9% of boys and 7.8% of girls consumed a minimum acceptable diet (MAD), and 6.2% of the boys and 7.8% of the girls met the minimum dietary diversity (MDD) cut-off.

**Table 2:** Infant and young child feeding practices in Oromiya and SNNP^a^ regions of Ethiopia, 2015

	Total population	Boys	Girls	*p*-value
	*n* = 2036	*n* = 1049	*n* = 987	
**IYCF indicators**				
Initiation of BF < 1 h %	75.7	76.9	74.5	0.196
Currently breastfeeding (CBF) %	99.7	99.8	99.5	0.275
Exclusive BF (EBF) at least 6 m %	77.6	77.0	78.2	0.524
EBF in months, mean (SD)	5.9 ± 1.2	5.9 ± 1.4	5.8 ± 0.8	0.344
**Introduction to soft and semi-solid foods**				
Age (months) CF introduced^b^, mean (SD)	5.9 ± 1.2	5.9 ± 1.3	6.0 ± 1.2	0.206
Introduction CF (6–8 months)^c^%	87.2	87.3	87.0	0.894
Minimum dietary diversity (MDD)^d^%	7.0	6.2	7.8	0.164
Minimum meal frequency (MMF)^e^%	75.3	74.8	75.9	0.607
Minimum acceptable diet (MAD)^f^%	6.8	5.9	7.8	0.095

^a^Southern Nations, Nationalities, and Peoples; CBF: currently breastfeeding (at the time of the survey); EBF: exclusive breastfeeding; IBF: initiation of breastfeeding; MDD: minimum dietary diversity; MMF: minimum meal frequency; MAD: minimum acceptable diet

^b^*n* = 1998: Boys *n* = 1031, Girls *n* = 967

^c^Received CF: children from 6 to 8 months who received solid, semi-solid, or soft foods in addition to breastfeeding

^d^Minimum dietary diversity: consumption of 4 or more food groups from the 7 food groups, namely: grains, roots, and tubers; legumes and nuts; dairy products (milk, yogurt, cheese); flesh foods (meat, fish, poultry, liver/organ meats); eggs; vitamin A-rich fruits and vegetables; and other fruits and vegetables

^e^Minimum meal frequency: consumption of 2 or more (at age 6–8 months), 3 or more (at age 9–23 months) solid or semi-solid feeds for breastfeeding children, or 4 or more solid or semi-solid or milk feeds for non-breastfeeding children at age 6–23 months

^f^Minimum acceptable diet: a combination of minimum dietary diversity and meal frequency

We examined gender differences in the consumption of seven selected food groups (Table 3). Most children (> 76%) had consumed cereals and roots/tubers during the previous 24 h. The consumption of legumes/nuts and eggs was low but increased slightly with age, with the highest consumption of legumes/nuts in boys aged 9–11 months (45.4%); this was significantly higher than the consumption of legumes/nuts in girls of that same age. The use of fruits and vegetables tended to be lower in the older boys compared to the girls (19.7% vs 25.0%, *p* = 0.057), whereas the consumption of eggs tended to be higher in older girls compared to boys (25.2% vs 20.5%, *p* = 0.099). The consumption of flesh foods was negligible. There were no significant differences in the consumption of any of the other food groups between boys and girls.

**Table 3:** Food group use (%) in young children in Oromiya and SNNP^a^ regions of Ethiopia, 2015

	6–8 months (*n* = 1139)			9–11 months (*n* = 923)		
Food groups^††^	Boys	Girls	*p*-value^‡^	Boys	Girls	*p*-value^‡^
	*n* = 562	*n* = 551		*n* = 487	*n* = 436	
Cereals and roots/tubers	76.9	78.8	0.471	90.1	89.9	0.913
Legumes and nuts	26.5	28.7	0.422	45.4	38.5	0.039
Dairy	32.9	34.7	0.568	31.6	33.7	0.527
Flesh foods	0	0	-	0.2	0	1.000
Eggs	18.9	20.5	0.498	20.5	25.2	0.099
Vitamin A-rich fruits and vegetables	4.6	4.9	0.888	4.3	5.0	0.641
Other fruits and vegetables	19.0	20.5	0.548	19.7	25.0	0.057

^a^Southern Nations, Nationalities, and Peoples

^†^^†^Calculated based on consumption of the food group in the previous 24 h

^‡^Chi-square tests were used to compare between boys and girls

Table 4 describes the factors associated with stunting and wasting. Gender was a significant determinant of nutritional status (odds ratio (OR) for boys vs girls: 1.91 (95%CI 1.48, 2.46) for stunting and OR 1.51 (95%CI 1.06, 2.16) for wasting), showing boys more affected than girls. In addition to gender, of the 18 characteristics investigated, the following were significantly associated with stunting: region of residence, non-exclusive breastfeeding, untimely introduction to CF, diarrhea during last 7 days, absence of consumption of legumes/nuts, lack of consumption of eggs, and maternal education. Determinants of wasting included region of residence, absence of consumption of legumes and nuts, absence of consumption of other fruits and vegetables, age of the mother, and education of the mother.

**Table 4:** Factors associated with stunting and wasting in Oromiya and SNNP^a^ regions of Ethiopia, 2015 (*n* = 2036)

**Variables**	**Stunting**^**b**^		**Wasting**^**c**^		
	**OR (95%CI)**^**d**^	***p*-value**	**OR (95%CI) **^**d**^		***p*-value**
Region SNNP	1.39 (1.08,1.78)	0.010	0.35 (0.24,0.52)		< 0.001
Oromiya	1		1		
**Child characteristics**					
Sex Male	1.91 (1.48,2.46)	< 0.001	1.51 (1.06,2.16)	0.022	
Female	1		1		
Age (month)	1.06 (0.98,1.14)	0.133	0.77 (0.54,1.09)	0.138	
IBF (Late)	1.22 (0.93,1.61)	0.158	1.00 (0.67,1.50)	1.000	
Before 1 h	1		1		
EBF (No)	1.56 (1.19,2.05)	0.001	1.03 (0.68,1.55)	0.908	
Yes	1		1		
Timely introduced to CF (No)	1.89 (1.38,2.60)	< 0.001	1.19 (0.73,1.94)	0.500	
Yes	1		1		
MDD (No)	1.30 (0.77, 2.20)	0.321	1.41 (0.65,3.08)	0.389	
Yes	1		1		
MMF (No)	1.17 (0.89,1.54)	0.276	0.90 (0.59,1.36	0.602	
Yes	1		1		
MAD (No)	1.27(0.75, 2.14)	0.372	1.38 (0.63,3.00)	0.423	
Yes	1		1		
Diarrhea last 7 days (Yes)	1.31 (1.01,1.70)	0.044	1.10 (0.75,1.61)	0.619	
No	1		1		
Consumed legumes and nuts (No)	1.42 (1.08, 1.86)	0.012	0.64 (0.45,0.91)	0.012	
Yes	1		1		
Consumed eggs (No)	1.41 (1.02,1.95)	0.038	0.82 (0.55,1.24)	0.345	
Yes	1		1		
Consumed other fruits and vegetables (No)	0.87 (0.65,1.17)	0.361	1.67 (1.02,2.75)	0.042	
Yes	1		1		
**Mother’s characteristics**					
Age of mother (< 25 years)	1.01 (0.79,1.30)	0.929	0.60 (0.42,0.85)	0.004	
> 25 years	1		1		
Education (illiterate)	1.32 (1.03,1.69)	0.027	1.42 (1.00,2.02)	0.049	
Literate	1		1		
Marital status (married)	0.61 (0.34,1.07)	0.086	0.49 (0.24,1.01)	0.054	
^4^	1		1		
Occupation (working mother)	0.75 (0.54,1.05)	0.095	1.46 (0.97,2.18)	0.069	
Housewife	1		1		
**HH characteristics**					
Basic drinking water (No)	0.83 (0.50,1.36)	0.458	0.47 (0.19,1.16)	0.099	
Yes	1		1		
Adequate sanitation (No)	0.78 (0.45,1.36)	0.378	1.55 (0.83,2.88)	0.171	
Yes	1		1		

^a^Southern Nations, Nationalities, and Peoples; OR: odds ratio; CI: confidence interval; IBF: initiation of breastfeeding; MDD: minimum dietary diversity; MMF: minimum meal frequency; MAD: minimum acceptable diet; EBF: exclusive breastfeeding; Timely introduced to CF: introduced to complementary food at 6–8 m; HH: household

^b^Stunted *n* = 302, Not stunted *n* = 1734

^c^Wasted *n* = 136, Not wasted *n* = 1900

^d^Univariate analyses were run using logistic regression with stunting or wasting as the dependent variable and each variable as an independent variable

^e^Single/separated/widowed/divorced

We further explored factors associated with stunting and wasting (Table 5) separately for boys and girls, taking age, sub-district/cluster, and characteristics of the mother and/or household into account. In boys, independently increased odds of stunting were observed for residing in SNNP, late initiation of breastfeeding, non-exclusive breastfeeding until age 6 months, untimely introduction to CF, absence of consumption of legumes/nuts, and an illiterate mother. Among girls, only untimely CF introduction, the absence of legumes/nut use, and non-use of eggs were significantly associated with the presence of stunting.

**Table 5:** Determinants of stunting and wasting in Oromiya and SNNP^a^ regions of Ethiopia, 2015

		**Boys,** *n* = 1049			**Girls, ***n* = 987			
**Variables**	**Stunting**		**Wasting**		**Stunting**		**Wasting**	
**AOR (95%CI)**		***p*-value**	**AOR (95%CI)**	***p*-value**	**AOR (95%CI)**	***p*-value**	**AOR (95%CI)**	***p*-value**
Region (SNNP)	2.00(1.41, 2.83)	< 0.001	0.45 (0.26, 0.78)	0.004	1.52 (0.98,2.36)	0.060	0.38 (0.19, 0.75)	0.006
**Child characteristics**								
Oromiya	1		1		1		1	
Age (month^b^)	1.07 (0.98,1.18)	0.140	1.11 (0.98, 1.27)	0.110	1.02 (0.90,1.15)	0.752	1.03(0.87,1.21)	0.755
IBF (Late)	1.46 (1.02,2.08)	0.037	-	-	0.88 (0.55, 1.41)	0.589	-	-
Before 1 h	1				1			
EBF(No)	1.66 (1.17,2.35)	0.004	-	-	1.28 (0.81,2.04)	0.294	-	-
Yes	1				1			
Timely introduced to CF								
(No)	2.14 (1.40,3.27)	< 0.001	**-**	-	1.85 (1.09,3.15)	0.024	-	-
Yes	1				1			
Diarrhoea last 7 days (Yes)	1.27 (0.90,1.78)	0.172	-	-	1.44 (0.94, 2.21)	0.096	-	-
No	1				1			
Consumed legumes and nuts (No)	1.45 (1.02, 2.06)	0.037	0.66 (0.41, 1.06)	0.088	1.86 (1.15, 3.00)	0.011	0.86(0.48,1.54)	0.605
Yes	1		1		1		1	
Consumed eggs (No)	1.21 (0.80,1.83)	0.363	-	-	1.76 (1.00, 3.08)	0.049	-	-
Yes	1				1			
Consumed other fruits and vegetables (No)	0.78 (0.53, 1.15)	0.209	1.40(0.74,2.66)	0.305	-	-	1.83 (0.81, 4.15)	0.150
Yes	1		1				1	
**Mother’s characteristics**								
Age of mother (< 25 years)	-	-	0.60 (0.37,0.97)	0.035	-	-	0.56 (0.31,1.00)	0.051
> 25 years			1				1	
Education (illiterate)	1.44 (1.05,1.97)	0.023	1.09(0.67,1.76)	0.741	1.22 (0.82,1.84)	0.329	1.55 (0.85, 2.80)	0.150
Literate	1		1		1		1	
Marital status (Married)	0.62 (0.29,1.31)	0.209	0.43 (0.17, 1.07)	0.070	0.44 (0.17, 1.11)	0.081	0.54(0.15,1.90)	0.335
^c^	1		1		1		1	
Occupation (^d^)	0.65 (0.43, 1.00)	0.051	1.15(0.67,1.98)	0.623	0.73 (0.42,1.28)	0.278	1.59 (0.84, 3.01)	0.155
House wife	1		1		1		1	
**HH characteristics**								
Basic drinking water (No)	-	-	0.30 (0.07, 1.26)	0.101	-	-	0.69(0.21,2.27)	0.537
Yes			1				1	
Adequate sanitation (No)	-	-	0.97(0.37, 2.53)	0.955	-	-	2.02 (0.86, 4.74)	0.106
Yes			1				1	

^a^Southern Nations, Nationalities, and Peoples; AOR: adjusted odds ratio; CI: confidence interval; IBF: initiation of breastfeeding; EBF: exclusive breastfeeding; Timely introduced to CF: introduced to complementary food at 6–8 m; HH: household

The adjustment was made for age, cluster (a study sub-district), and mother’s characteristics

^b^Adjusted for cluster only and mother’s characteristics

^c^Single/separated/widowed/divorced

^d^Working mother

Regarding wasting, for both boys and girls, the region of residence and age of the mother were the only significant independent risk factors, with lower odds of wasting when residing in SNNP (compared to Oromiya) and when having a mother < 25 years of age (Table 5). Consumption of legumes and mother’s marital status are not significant (*p* < 0.05) but they tended to be significant determinants for wasting in boys (*p* < 0.10) but not in girls, whereas the absence of adequate sanitation tended to be significant in girls but not in boys.

The interaction terms between gender and all main determinants were tested in additional logistic regression analyses. There was an indication of an interaction between early initiation of breastfeeding and gender (*p* = 0.128), suggesting that the association between initiation of breastfeeding and stunting was different between boys and girls. No clear interactions with sex were observed for the other variables, neither for stunting nor for wasting (see Additional file 1).

Determinants were also investigated in relation to the LAZ z score and WLZ z score as continuous outcome variables (see Additional file 2). Boys had a lower mean of LAZ z score than girls, and LAZ z score decreased with age, was lower in those with the untimely introduction of CF, not consuming legumes and nuts and not consuming eggs, and having a mother or caregiver < 25 years of age. WLZ z score was lower in Oromiya in boys; decreased with age; and was lower in those with no MDD, in the presence of diarrhea, in older mothers or caregivers, in non-working mothers, and from a household with inadequate sanitation.

## Discussion

Our analyses show that gender differences in stunting and wasting exist in Ethiopian infants aged 6–11 months, with boys being 1.9 times more likely to be stunted and 1.5 times more likely to be wasted than girls. Risk factors for stunting and wasting are not significantly different between boys and girls, although small differences exist. Region of residence, untimely initiation of breastfeeding, not-exclusive breastfeeding at age 6 months, and low maternal education are significant risk factors for stunting in boys. Untimely introduction to complementary food and low consumption of legumes/nuts are significant risk factors for stunting in both boys and girls, and only in girls is low egg consumption associated with stunting. Region of residence and mother’s age are the significant independent predictors of wasting in both sexes.

The gender differences that we observed in nutritional status are consistent with findings from the northern part of Ethiopia [20] as well as from some other Sub-Saharan African countries [10, 21–25]. A meta-analysis of 16 demographic and health surveys from 10 Sub-Saharan African countries in 2007 also shows that male children were 1.16 times more likely to be stunted than females [26]. A recent meta-analysis of data on children (6–59 months) from 84 countries in 2018 also reports a similar trend of a significantly higher prevalence of stunting (34.3% vs 31.7%) and wasting (9.5% vs 8.1%) among boys compared to girls [27].

Although poor IYCF practices were observed in both boys and girls, our multivariate analysis shows that risk factors for stunting are not completely similar for each gender. Variation in the initiation of breastfeeding and exclusive breastfeeding are significant independent determinants of stunting in boys, but not in girls, suggesting a higher vulnerability to poor feeding practices among boys compared to girls in the first months of life. In contrast, timely introduction to CF is a common determinant of stunting for both boys and girls.

Several possible explanations are reported concerning gender differences in nutritional status. First of all, these differences might result from biological differences that could be independent of infant feeding patterns [9]. For instance, boys are more susceptible to infectious diseases [26] and show higher biological fragility in the first year of life [28]. However, the underlying mechanisms for the biological difference are poorly understood [26]. Secondly, a study in Madagascar [29] found that gender differences in stunting tend to vary with age, with males more likely to become stunted in the first year, whereas females are more likely to become stunted in the second year of life [30]. Our study included only children in the first year of life, and this could partly explain the higher stunting rates in boys.

The high morbidity observed in our study may be associated with a high number of illiterate mothers in the study regions. The finding of the association between increased risk of morbidity and mother’s illiteracy is consistent with findings of studies conducted in rural Ethiopia [31] and Tanzania [32]. Our results show that the mother’s literacy status and maternal occupation are two of the independent determinants of stunting in boys but not in girls. Studies in Southern Ethiopia [33], Mozambique [25], and Bangladesh [34] also found the mother’s literacy status to be associated with stunting. Literacy status may be indicative of a mother’s better knowledge and awareness of child nutrition and may therefore result in relatively better feeding practices [34].

One of the main findings of this study is that the region of residence is a determinant of stunting and wasting independent of the other determinants. We observed regional differences in stunting and wasting, where stunting is higher (OR 1.39) but wasting lower (OR 0.35) in SNNP compared to Oromiya. In the Ethiopia 2016 DHS, a similar trend was observed for stunting (38.6% vs 36.5%) and for wasting (6.0% vs 10.6%) in SNNP versus Oromiya, respectively, for children under 5 years of age [2]. According to Teklie et al. (2019), the odds of being wasted in SNNP and Oromiya were 0.365 and 0.727 times lower compared to the Tigray region respectively, indicating the lower odds of SNNP compared to Oromiya [35]. A recent meta-analysis conducted in Sub-Saharan Africa also shows regional differences in stunting patterns and suggests the importance of contextualizing appropriate nutrition interventions [36, 37].

A limitation of this study is that it uses the baseline data of an intervention study, with one of the inclusion criteria being WLZ z scores > -3 SD. This means that first, we cannot exclude the possibility that the results, of for example differences between regions, might have been different if severely stunted children had been included. Second, we are unable to account for unknown confounding variables. Third, birth weight data were not collected in this study, mainly because it was beyond the scope of the study. Hence, the effect of low birth weight on nutritional status cannot be measured. Fourth, due to the cross-sectional nature of the study, we could only discuss the association rather than causation. Fifth, our study did not address infection-driven malnutrition. However, at the time of data collection, there was no significant difference in the presence of morbidity between female and male children. Lastly, another important limitation of our study is that the generalizability of our finding is limited to young children (6–11 months), which might result in different risk factors in other age groups.

The main strength of the study is that it involves a large sample size that represents the target population in the two large regions among the three largest regions of the country namely Amhara, Oromiya and SNNP and an area where nutritional improvements are needed. Secondly, the study team underwent 9 days of intensive training, including standardization of data collectors and pilot testing of the questionnaires, which helped to refine the questionnaires and avoid questions that might lead to biased answers [38]. This reduced the measurement error that can occur during data collection if measurements are collected differently in exposure and outcome. To our knowledge, this is the first study to assess gender differences in determinants of nutritional status in these regions in Ethiopia with such a large sample size (*n* = 2036).

Even though the selected districts are UNICEF’s Community-Based Nutrition (CBN) districts, which presumably have better Infant and Young Child Nutrition (IYCN) programs, the observed IYCF practices are suboptimal; this partly suggests that these districts have been correctly targeted, as the worst-off districts are more eligible for CBN interventions. However, it also suggests the need for more efforts to strengthen the on-going IYCN programs within the CBN districts. An ethnographic study on gender-related maternal beliefs and attitudes regarding IYCF practices would help to better clarify the underlying causes of the observed gender differences and the differences in vulnerability of the two sexes during infancy. In addition, there is emerging evidence that early life nutrition may affect the development of chronic diseases differently in boys than in girls [39]. For instance, birth weight has been found to predict the risk of insulin resistance later in life in men, but not in women [39]. Therefore, the observed gender differences in early life nutrition may have long-term health implications and need to be addressed [39]. Despite the inclusion of relatively healthy children in the study, we do not expect any problems regarding the generalizability of the recommended IYCF practices to other areas of the country, especially to a similar age group population. As stated in the report of the national survey; these age group children’s characteristics are similar to those in other sub-districts in Ethiopia [13]. However, the fact that there exists a difference in sample size in both sex; might result in variability of some of the estimates thus further study is needed with a larger sample size.

## Conclusion

In conclusion, the results of this study show that gender differences in nutritional status exist in Ethiopia: girls have a better nutritional status compared to boys during the first year of life. Determinants of stunting and wasting are largely similar between the sexes, although poor breastfeeding practices in the first 6 months of life seemed to affect stunting more in boys than in girls. The observed gender and regional differences suggest the need for region-specific intervention plans emphasizing gender-sensitive guidance on optimum IYCF practices. Our results suggest the need for addressing child undernutrition in early infancy by promoting appropriate breastfeeding practices, for instance, early initiation of breastfeeding with emphasis on exclusive breastfeeding (EBF) until the child reaches 6 months. EBF and adequate dietary diversity of complementary feeding are important determinants of stunting in boys and girls. The findings of this study will contribute to the development of gender-sensitive behavior change intervention materials that would contribute to the effort to convince and guide mothers to introduce complementary foods in a timely fashion, regardless of the child’s gender.

## Supplementary Information

**Additional file 1.**

**Additional file 2.**

**Additional file 3.**

## Data Availability

The data collected and analyzed for this study are available from the corresponding author on reasonable request.

## Abbreviations

BF: Breastfeeding
CBF: Current breastfeeding
CF: Complementary food
CI: Confidence interval
cm: Centimeter
EBF: Exclusive breastfeeding
EPHI: Ethiopian Public Health Institute
LAZ: Length-for-age Z score
IBF: Initiation of breastfeeding
IYCF: Infant and young child feeding
IYCN: Infant and young child nutrition
MAD: Minimum acceptable diet
MDD: Minimum dietary diversity
MMF: Minimum meal frequency
NI: Nutrition International
SD: Standard deviation
SNNPR: South Nations, Nationalities, and Peoples Region
SPSS: Statistical Package for the Social Sciences
UNICEF: United Nation Children’s Fund
WASH: Water, Sanitation, and Hygiene
WLZ: Weight-for-length z score
WHO: World Health Organization

## References

[1] UNICEF, WHO (2021). International Bank for Reconstruction and Development/The World Bank. Levels and trends in child malnutrition: key findings of the 2021 edition of the joint child malnutrition estimates.

[2] CSA (2016). Ethiopia demographic and health survey 2016.

[3] Herrador Z, Sordo L, Gadisa E, Moreno J, Nieto J, Benito A (2014). Cross-sectional study of malnutrition and associated factors among school aged children in rural and urban settings of Fogera and Libo Kemkem districts, Ethiopia. PLoS One.

[4] Black RE, Allen LH, Bhutta ZA, Caulfield LE, De Onis M, Ezzati M (2008). Maternal and child undernutrition: global and regional exposures and health consequences. Lancet.

[5] Richard SA, Black RE, Gilman RH, Guerrant RL, Kang G, Lanata CF (2013). Diarrhea in early childhood: short-term association with weight and long-term association with length. Am J Epidemiol.

[6] Ruel MT, Menon P (2002). Child feeding practices are associated with child nutritional status in Latin America: innovative uses of the demographic and health surveys. J Nutr.

[7] Pearce J, Taylor M, Langley-Evans S. Timing of the introduction of complementary feeding and risk of childhood obesity: a systematic review. Int J Obes (Lond). 2013; 37:1295–306. Available from: https://www.nature.com/articles/ijo201399.pdf. 10.1038/ijo.2013.99 23736360

[8] Hop LT, Gross R, Giay T, Sastroamidjojo S, Schultink W, Lang NT (2000). Premature complementary feeding is associated with poorer growth of Vietnamese children. J Nutr.

[9] Bork KA, Diallo A (2017). Boys are more stunted than girls from early infancy to 3 years of age in rural Senegal. J Nutr.

[10] Bukusuba J, Kaaya AN, Atukwase A (2017). Predictors of stunting in children aged 6 to 59 months: a case–control study in Southwest Uganda. Food Nutr Bull.

[11] Tumilowicz A, Habicht J-P, Pelto G, Pelletier DL (2015). Gender perceptions predict sex differences in growth patterns of indigenous Guatemalan infants and young children–3. Am J Clin Nutr.

[12] Samuel A, Brouwer ID, Feskens EJM, Adish A, Kebede A, De-Regil LM (2018). Effectiveness of a Program Intervention with Reduced-Iron Multiple Micronutrient Powders on Iron Status, Morbidity and Growth in Young Children in Ethiopia. Nutrients.

[13] EPHI (2013). Ethiopia national food consumption survey.

[14] de Onis M, Onyango AW, Van den Broeck J, Chumlea WC, Martorell R (2004). Measurement and standardization protocols for anthropometry used in the construction of a new international growth reference. Food Nutr Bull.

[15] WHO (2010). Indicators for assessing infant and young child feeding practices. part 2 measurement.

[16] WHO (2011). WHO Anthro (version 3.2. 2, January 2011) and macros (2011).

[17] WHO (2008). Indicators for assessing infant and young child feeding practices. part 1 definitions.

[18] UNICEF. Post-2015 WASH targets and indicators: outcomes of an expert consultation2015. Available from: https://www.unicef.org/wash/files/4_WSSCC_JMP_Fact_Sheets_4_UK_LoRes.pdf.

[19] Selvin S (2004). Statistical analysis of epidemiologic data.

[20] Alemu ZA, Ahmed AA, Yalew AW, Birhanu BS, Zaitchik BF (2017). Individual and community level factors with a significant role in determining child height-for-age Z score in East Gojjam Zone, Amhara Regional State, Ethiopia: a multilevel analysis. Arch Public Health.

[21] Chirande L, Charwe D, Mbwana H, Victor R, Kimboka S, Issaka AI (2015). Determinants of stunting and severe stunting among under-fives in Tanzania: evidence from the 2010 cross-sectional household survey. BMC Pediatr.

[22] Linnemayr S, Alderman H, Ka A (2008). Determinants of malnutrition in Senegal: individual, household, community variables, and their interaction. Econ Hum Biol.

[23] Pongou R, Ezzati M, Salomon JA. Household and community socioeconomic and environmental determinants of child nutritional status in Cameroon. BMC Public Health. 2006; 6(1):98. Available from: http://www.biomedcentral.com/1471-2458/6/98. 10.1186/1471-2458-6-98 PMC1523206 16618370

[24] Ali Z, Saaka M, Adams A-G, Kamwininaang SK, Abizari A-R (2017). The effect of maternal and child factors on stunting, wasting and underweight among preschool children in Northern Ghana. BMC Nutr.

[25] García Cruz L, González Azpeitia G, Reyes Súarez D, Santana Rodríguez A, Loro Ferrer J, Serra-Majem L. Factors associated with stunting among children aged 0 to 59 months from the Central Region of Mozambique. Nutrients. 2017; 9(5):491. Available from: http://www.mdpi.com/2072-6643/9/5/491. 10.3390/nu9050491 PMC5452221 28498315

[26] Wamani H, Åstrøm AN, Peterson S, Tumwine JK, Tylleskär T (2007). Boys are more stunted than girls in Sub-Saharan Africa: a meta-analysis of 16 demographic and health surveys. BMC Pediatr.

[27] Khara T, Mwangome M, Ngari M, Dolan C (2018). Children concurrently wasted and stunted: a meta-analysis of prevalence data of children 6–59 months from 84 countries. Matern Child Nutr.

[28] Kraemer S (2000). The fragile male. BMJ.

[29] Rakotomanana H, Gates GE, Hildebrand D, Stoecker BJ. Determinants of stunting in children under 5 years in Madagascar. Matern Child Nutr. 2016:e12409. Available from: 10.1111/mcn.12409. 10.1111/mcn.12409 PMC6865998 28032471

[30] Adair LS, Guilkey DK (1997). Age-specific determinants of stunting in Filipino children. J Nutr.

[31] Girma M, Astatkie A, Asnake S (2018). Prevalence and risk factors of tungiasis among children of Wensho District, Southern Ethiopia. BMC Infect Dis.

[32] Mshida HA, Kassim N, Mpolya E, Kimanya M (2018). Water, sanitation and hygiene practices associated with nutritional status of under-five children in semi-pastoral communities Tanzania. Am J Trop Med Hyg.

[33] Eshete H, Abebe Y, Loha E, Gebru T, Tesheme T (2017). Nutritional status and effect of maternal employment among children aged 6–59 months in Wolayta Sodo town, southern Ethiopia: a cross-sectional study. Ethiop J Health Sci.

[34] Choudhury N, Raihan MJ, Sultana S, Mahmud Z, Farzana FD, Haque MA, et al. Determinants of age-specific undernutrition in children aged less than 2 years the Bangladesh context. Matern Child Nutr. 2017; 13(3). Available from: 10.1111/mcn.12362. 10.1111/mcn.12362 PMC6865922 27731545

[35] Tekile AK, Woya AA, Basha GW (2019). Prevalence of malnutrition and associated factors among under-five children in Ethiopia: evidence from the 2016 Ethiopia Demographic and Health Survey. BMC Res Notes.

[36] Akombi BJ, Agho KE, Merom D, Renzaho AM, Hall JJ (2017). Child malnutrition in sub-Saharan Africa: a meta-analysis of demographic and health surveys (2006–2016). PLoS One.

[37] Osgood-Zimmerman A, Millear AI, Stubbs RW, Shields C, Pickering BV, Earl L, et al. Mapping child growth failure in Africa between 2000 and 2015. Nature. 2018; 555:41. Available from: 10.1038/nature25760. 10.1038/nature25760 PMC6346257 29493591

[38] Bowden A, Fox-Rushby J, Nyandieka L, Wanjau J (2002). Methods for pre-testing and piloting survey questions: illustrations from the KENQOL survey of health-related quality of life. Health Policy Plan.

[39] Adair LS. Early nutrition conditions and later risk of disease. In: Popkin BCaBM, editor. The Nutrition Transition. London Elsevier; 2002. p. 129–45.

