# Supplementary material for: Gender differences in nutritional status and determinants among infants (6–11 m): a cross-sectional study in two regions in Ethiopia
Source: BMC Public Health. 2022 Feb 26;22:401. doi: 10.1186/s12889-022-12772-2 (PMC8881837; doi:10.1186/s12889-022-12772-2)
Supplement: Supplementary file 1 — Additional file 1. [file 12889_2022_12772_MOESM1_ESM.docx]

Additional file 1. Interactions terms between gender and determinants of undernutrition in Oromiya and SNNP^†^ regions, Ethiopia, 2015^1^

|  | | **Stunting** | | **Wasting** | |
| --- | --- | --- | --- | --- | --- |
| **Variables** | | **Exp(β) (95%CI)** | ***p*-value** | **Exp(β) (95%CI)** | ***p*-value** |
| Region (Oromiya^††^) | | 0.76(0.45,1.27) | 0.289 | 1.50(0.68,3.29) | 0.317 |
| **Child characteristics** | | |  |  |  |
| Age (month) | | 1.04(0.90,1.21) | 0.583 | 0.93(0.75,1.15) | 0.494 |
| IBF | | 1.57(0.88,2.82) | 0.128 | ‡ |  |
| EBF | | 1.27(0.71,2.25) | 0.418 | ‡ |  |
| MDD | | ‡ |  | ‡ |  |
| MMF | | ‡ |  | ‡ |  |
| MAD | | ‡ |  | ‡ |  |
| Timely introduced to CF | | 1.12(0.58,2.18) | 0.732 | ‡ |  |
| Diarrhoea last 7 days | | 0.90(0.52,1.54) | 0.689 | ‡ |  |
| Consumed legumes and nuts | | 0.79(0.45,1.41) | 0.429 | 1.26(0.61,2.59) | 0.537 |
| Consumed eggs | | 0.70(0.35,1.38) | 0.302 | ‡ |  |
| Consumed other fruits and vegetables | | ‡ |  | 0.99(0.43,2.27) | 0.987 |
| **Mother’s characteristics** | | |  |  |  |
| Age of mother (>25 year^††^) | ‡ | |  | 1.12(0.55,2.28) | 0.764 |
| Education | | 1.18(0.71,1.96) | 0.532 | 1.34(0.65,2.76) | 0.427 |
| Marital status | | 1.20(0.37,3.84) | 0.763 | 1.26(0.28,5.74) | 0.765 |
| Occupation | | 0.84(0.42,1.67) | 0.617 | 1.47(0.65,3.33) | 0.361 |
| **HH characteristics** | |  |  |  |  |
| Basic drinking water | | ‡ |  | 0.47(0.07,2.99) | 0.421 |
| Adequate sanitation | | ‡ |  | 0.45(0.13,1.60) | 0.220 |

^†^ Southern Nations, Nationalities, and Peoples; Exp(β): odds ratio; CI: confidence Interval; IBF: initiation of breastfeeding; EBF: exclusive breastfeeding; MDD: minimum dietary diversity; MMF: minimum meal frequency; MAD: minimum acceptable diet; Timely introduced to CF: introduction to complementary food at 6–8 m; HH: household

**^1^** Interaction was evaluated (each variable+ gender + gender X each variable) using logistic regression and results for testing interaction terms between gender and other determinants of stunting and wasting are presented; a *p*<0.2 was considered relevant for interaction terms; n=2036

^††^ Reference category

‡ Not included in Tables 5
