# Supplementary material for: Gender differences in nutritional status and determinants among infants (6–11 m): a cross-sectional study in two regions in Ethiopia
Source: BMC Public Health. 2022 Feb 26;22:401. doi: 10.1186/s12889-022-12772-2 (PMC8881837; doi:10.1186/s12889-022-12772-2)
Supplement: Supplementary file 2 — Additional file 2. [file 12889_2022_12772_MOESM2_ESM.docx]

Additional file 2. Multiple predictors^1^ of LAZ and WLZ in Oromiya and SNNP^†^ regions of Ethiopia, 2015

|  | **Lengthfor Age (LAZ)** | | **Weight for Length (WLZ)** | |
| --- | --- | --- | --- | --- |
| **Variables** | **β(SE)** | ***P* value** | **β(SE)** | ***P* value** |
| Intercept | -0.31(0.16) | 0.046 | -0.14(0.14) | 0.315 |
| Region (SNNP^††^) | 0.07(0.06) | 0.249 | -0.23(0.06) | <0.001 |
| **Child characteristics** | |  |  |  |
| Sex (Female^††^) | -0.42(0.06) | <0.001 | -0.11 (0.05) | 0.017 |
| Age (month, 11 m^††^) |  |  |  |  |
| 6 months | 0.39(0.11) | <0.001 | 0.35(0.09) | <0.001 |
| 7 months | 0.71 (0.12) | <0.001 | 0.23(0.10) | 0.015 |
| 8 months | 0.51 (0.12) | <0.001 | 0.11(0.10) | 0.263 |
| 9 months | 0.47 (0.12) | <0.001 | 0.08(0.09) | 0.381 |
| 10 months | 0.17 (0.12) | 0.156 | -0.02(0.09) | 0.860 |
| IBF (before 1 hr^††^) | -0.03(0.07) | 0.647 | ‡ |  |
| EBF (yes^††^) | -0.15(0.08) | 0.069 | ‡ |  |
| MDD (yes^††^) | -0.13(0.13) | 0.318 | -0.26(0.10) | 0.007 |
| MMF (yes^††^) | ‡ |  | -0.07(0.06) | 0.234 |
| Timely introduced to CF (yes^††^) | -0.45(0.10) | <0.001 | ‡ |  |
| Diarrhoea last 7 days (yes^††^) | ‡ |  | 0.23(0.06) | <0.001 |
| Consumed legumes and nuts (yes^††^) | -0.17(0.07) | 0.013 | ‡ |  |
| Consumed eggs (yes^††^) | -0.17(0.08) | 0.032 | ‡ |  |
| Consumed other fruits and vegetables (yes^††^) | ‡ |  | 0.001(0.07) | 0.983 |
| **Mother’s characteristics** | |  |  |  |
| Age of mother (>25 years^††^) | -0.14(0.06) | 0.019 | 0.10(0.05) | 0.047 |
| Education (literate^††^) | ‡ |  | -0.06(0.05) | 0.237 |
| Marital status^2††^ | ‡ |  | ‡ |  |
| Occupation^3††^ | ‡ |  | 0.16(0.06) | 0.008 |
| **HH characteristics** |  |  |  |  |
| Basic drinking water (yes^††^) | ‡ |  | ‡ |  |
| Adequate sanitation (yes^††^) | ‡ |  | -0.21(0.10) | 0.037 |

^†^ Southern Nations, Nationalities, and Peoples; β: unstandardized coefficients; SE: standard error; IBF: initiation of breastfeeding; EBF: exclusive breastfeeding; MDD: minimum dietary diversity; MMF: minimum meal frequency; Timely introduced to CF: introduced to complementary food at 6–8 m; HH: household

^1^ Variables included in multiple linear regression are based on simple regression with *p*<0.20, n=2035

^2^ Single/separated/widowed/divorced

^3^ Housewife

^††^ Reference category

‡ *p*>0.2 and not included in the model
