# Supplementary material for: Gender differences in nutritional status and determinants among infants (6–11 m): a cross-sectional study in two regions in Ethiopia
Source: BMC Public Health. 2022 Feb 26;22:401. doi: 10.1186/s12889-022-12772-2 (PMC8881837; doi:10.1186/s12889-022-12772-2)
Supplement: Supplementary file 3 — Additional file 3. [file 12889_2022_12772_MOESM3_ESM.docx]

**Gender differences in nutritional status and determinants among infants (6–11m): a cross-sectional study in two regions in Ethiopia**

Aregash Samuel*^1^, Saskia J.M Osendarp^2^, Edith J.M Feskens^2^, Azeb Lelisa^3^, Abdulaziz Adish^3^, Amha Kebede^1^, Inge D. Brouwer^2^

^1^Ethiopian Public Health Institute (EPHI), ^2^ Division of Human Nutrition and Health, Wageningen University, ^3^Nutrition International, Ethiopia

*Corresponding author: Dr Aregash Samuel, email: [aregash.ephi@gmail.com](mailto:aregash.ephi@gmail.com), (AS)

**Questionnaire for data collection**

**1.1 Date of Interview:** | |__|/| |__|/__| |__| |

**1.2 Enumerator Name:_______________________________ Signature**: _________

**1.3 Supervisor/Coordinator Name: Signature:**

**1.4 Region ID______ 1= Oromia; 2= SNNPR**

**1.5 Zone ………………………………1.6 Woreda: …………………………**

**1.7 Kebele: ………………. 1.8 Name of the HP……………………….**

**SECTION 1: ADMISSION QUESTIONNAIRE**

**Please ask all questions about the child and write the responses given in the space provided for**

**1.9** Name of the Child (please write full name) ……………………………..……………………………….

**1.10** Unique HH Code: ……………………………..………………………………………………….

**1.11** Child’s unique ID*: |R |Z |W|Kebele |Kebele |HH |HH| Child no| | | | | | | | | |

**1.12** Name of Caregiver/Mother:

**1.13** Sex of the child? ***CIRCLE*** 1=Male 2=Female

**1.14** Date of birth

| | |/|__| |/| | |

(Day/month/year)

Verification source:___________________________________________

**1.15** Age of the child? (*in months*)** **| | |**

**1.16** Weight (Kg) of the child Measurement 1. | | |.| |kg

Measurement 2. | | |.| |kg

**1.17** Height (cm) of the child Measurement 1. | | | |.| |cm

Measurement 2. | | | |.| |cm

**1.18** MUAC (cm) of the child Measurement 1. | | |.| |cm

Measurement 2. | | |.| |cm

1= Below -3SD

**1.19** WFH z-score

**1.20 MUAC cut off point** *(>11.0 cm) CIRCLE*

2= Equal or Above -3SD and below -2SD

3= Equal or above -2SD

1=Yes 2=No

**1.21** Oedema(Confirmed) 1= Yes 2=No

**1.22** Severe anaemic (by

observation)

1= Yes 2=No

**Note: If the child falls between>-3SD and <3SD Z-score and MUAC is yes, continue with the questions below. If no, end here.**

| **1.23** Mention the illnesses/diseases/conditions your child has suffered in the last 7 days  ***CIRCLE ALL THAT APPLY*** | 1 = Fever  1 = Fever/malaria  2 = Vomiting  3 = Diarrhoea (three or more times loose and watery stools per day, or blood in stool)  4 = Cough with short, difficult, faster breathing than usual  5 = No appetite as usually when compared with usual intake  6 = Skin Infections  7= Other  8 = No Illness |
| --- | --- |
| **1.24** Mention the MEDICATION OR TREATMENT your child has received in the last 6 months  *CIRCLE ALL THAT APPLY* | 1 = De-worming  2 = Treatment of malaria, flu, cough  3 = Measles immunization  4 = Vitamin A supplementation in previous 6 months  5= Use of antibiotics  6 = Others  7 = No medication/treatment |
| **Note: If the child had any of the illnesses/diseases/conditions, or under medication/treatment (except no 5) and looks better currently, continue with the following questions. However if the child has taken antibiotics during the last 3 month, thank the mother and stop here.** | |
| **1.25** Does your child has ever had any chronic health problem? | 1=Yes 2=No  If yes, please mention_ |
| **1.26** Mention if you (care giver or mother) are planning to stay in the study area for one year (at least). | 1=Yes 2=No |
| **1.27** Referred child: | 1=Yes 2=No |
| **1.28** Admitted to the study: | 1=Yes 2=No |

* To be filled after admission

**Child needs to be between 6 months and 11.99 months old at the day of recruitment

| **SECTION 2: MOTHER/CAREGIVER INFORMATION** | |
| --- | --- |
|  |  |
| **Please ask all questions about child and write the responses given in the space provided for** | |
| **2.1.1** Mother/Caregiver name | 1. _____________________ |
| **2.1.2** Respondent relationship to the target child | 1= Mother 2 = Care taker |
| **2.1.3** Age of mother/care taker | \|__\|__\| years |
| **2.1.4** # of children by this mother/care taker (Parity) | \|__\|__\| |
| **2.1.5** Is mother/caretaker pregnant or breast-feeding? ***CIRCLE ONE*** | 1. Pregnant  2. Breast feeding (lactating)  3. Pregnant and breast feeding  4. None of the above |
| **2.1.6** Educational level of mother/care taker ***CIRCLE ONE*** | 1. Illiterate  2. None formal education  3. Grade 1 – 4  4. Grade 5 – 8  5. High School 9 – 10  6. 10 and above  7. Other(Specify) |
| **2.1.7** Marital status ***CIRCLE ONE*** | 1. Single  2. Married  3. Separated  4. Widowed  5. Divorced |
| **2.1.8** Do you have farmland? ***CIRCLE ONE*** | 1 = Yes 2 = No. ***If yes, continue with question 2.1.9; if no, to 2.1.13*** |
| **2.1.9** If yes, for question 2.1.8, what is its estimated size (Indicate unit of measure) |  |
| **2.1.10** Does your household produce wheat? | 1 = Yes 2 = No. ***If yes, continue with question 2.1.11; if no, to 2.1.13*** |
| **2.1.11** How much hectar of wheat does your household cultivate per year? | 1. [ ] local unit; [ ]Hectar  (if the mother tells local measure, write which local unit and convert it in to hectar)  2. Do not know |
| **2.1.12** How much yield (in kg) does your household get per year? | 1. [ ] local unit; [ ] kg;  (if the mother tells local measure, write which local unit and convert it in to kg)  2. Do not know |
| 2.1.13. How many days and how often in a day (starting from yesterday) in the previous week did you prepare or consume meals based on wheat (including bread) in your household? | 1. days  2. times per day |
| 2.1.14.What is your main (usual) occupation? ***CIRCLE*** | 1. Housewife  2. Civil servant  3. Farmer  4. Petty trader  5. Daily labourer  6. Other (Specify)__________ |
| 2.1.15 What is the main source of drinking water for your household? | *A Piped water/supply water: A.1- Public tap*  *A 2 – Private tap*  *B Water from spring:*  *B.1 - Protected spring*  *B.2 - Unprotected spring*  *C. Water from Dug well:*  *C.1 - Protected well*  *C.2 - Unprotected well*  *D. Water form borehole*  *D.1 - Borehole in yard/plot*  *D.2 - Public borehole*  *E. Surface water*  *F. Pond/lake/River/stream/spring/Dam*  *G. Tanker truck*  *H. Rain water* |
| 2.1.16. What kind of toilet facility does your household have? | 1. Flush toilet  2. Pit latrine  3. No facility/bush/field  4. Other(specify) |
| 2.1.17. Tell me please what your house has from the following list:  ***Multiple answers are possible*** | 1. Electricity  2. Watch/clock  3. Television  4. Radio  5. Refrigerator |
| 2.1.18 How many of the following animals do you keep?  *RECORD “00” if the HH don’t have the particular*  *animal* | cows, oxen , or bulls __\|__\|  Goats \| \|__\|  Chickens __\|__\|  Sheep \| \|  Horses, donkey or mule \|__\| \|  Camels \| \|__\|  Beehives \| \| \| |
| 2.1.19 Tell me the number of your family members | 1. Below 2 years of age\| \|  2. 2-5years of age \|_ \|  3. Above 5 years \|_ \| |

| **SECTION III: INFANT NUTRITION** | |
| --- | --- |
| **Please ask all questions about the Child and write the responses given in the space provided for** | |
| **3.2.1** How long after birth did you breast fed? ***CIRCLE*** | 1.Immediately (with in an hr)  2. 1-12 hours  3.More than 12 hours  4. Don’t remember |
| 3.2.2 How many months did you exclusively breastfeed your child? (Feeding the child on only human breast milk and nothing else, not even water) | \|__\|__\| months |
| 3.2.3. What foods were introduced before the child reached 6 months of age? ***Only for non-exclusive Breastfed child.***  ***CIRCLE ALL THAT APPLY*** | 1. Water  2. Milk (other than breast milk)  3. Juice  4. Cereal Porridge  5. Tea  6. Others please specify------------------  7. Unknown |

| **SECTION IV–CHILD FEEDING AND 24 HR DIET RECALL** | | | | | | | | |
| --- | --- | --- | --- | --- | --- | --- | --- | --- |
| 4.3.1 Are you still breastfeeding your child? | | | | | 1= Yes 2= No | | | |
| 4.3.2 How many times did (name) breastfeed between yesterday sunrise and today sunrise? | | | | | \| _\| times | | | |
| 4.3.3 At what age of your child did you introduce  Liquid/solid foods? | | | | | \|__\|__\| months | | | |
| 4.3.4 How many times (meals) did (child’s name) eat  solid, semisolid or soft foods between yesterday  sunrise and today sunrise? (answer can include ‘zero’) | | | | | \|_\| times | | | |
| 4.3.5 Please describe the foods (meals , snacks and everything) that your child ate or drank yesterday (from sunrise until today sunrise) during the day and night, whether at home or outside the home. Start with the first food or drink of the morning.  The following spaces can be used to take brief notes on everything – food and beverage – that the child ate yesterday, with the time it was consumed. If used, this must be expanded into the following pages. (*use Ethiopian time) | | | | | | | | |
| Breakfast | | Snack | Lunch | Snack | | Dinner | | Snack |
|  | |  |  |  | |  | |  |
| 4.3.6. | Did your child eat anything (meal or snack) OUTSIDE of the home yesterday? CIRCLE and specify in the form (if the response is yes) | | | | | | 1= Yes 2= No | |
| 4.3.7. | Read: Now I would now like to ask you a few more detail questions about the foods given to the (child’s name) yesterday from sunrise until today sunrise. Ask by referring [4.3.5] | | | | | | | |

**24 HOUR DIET RECALL**

Time of day: 1=Morning (sunrise yesterday to ~5:30), 2=Afternoon (~5:30-sunset), 3= Evening/ night time (sunset to just before sunrise this morning)

**Order Time M/N Name of Food, Drink Source of food Description of food Food group Form of prep Code of prep**

**Code – SOURCE OF FOOD: Code – FORM OF PREP: Code – FOODGROUPS:**

1 = Home garden / wild (e.g. mango)

4 = Purchased

1= no change/ raw/ fresh (raw)

5 = Boiled, drained (boil,d)

9 = Other, no oil (no 1=grains, roots and tubers

5=eggs

2 = Obtained by trade

8 = Other 10= unknown

2=Fermented oil)

2=legumes and nuts

6=vitamin-A rich fruits

3 = Food aid or exchange for work

3 = Steamed (steam)

4 = Boiled, not drained (boil, nd)6 = Roasted/baked (bake)

7=Deep fried in oil (fry) 10 =unknown

3=dairy products (milk, yogurt, cheese) and vegetables

4=flesh foods (meat, fish, poultry and

liver/organ meats)

7=Other fruits and vegetables

8= Other, with oil (oil)

**Thank the mother/care taker and end here.**
